# Acute Effects of Ballistic and Non-ballistic Bench Press on Plyometric Push-up Performance

**DOI:** 10.3390/sports7020047

**Published:** 2019-02-18

**Authors:** David Bodden, Timothy J. Suchomel, Ally Lates, Nicholas Anagnost, Matthew F. Moran, Christopher B. Taber

**Affiliations:** 1 Department of Physical Therapy and Human Movement Science, Sacred Heart University, Fairfield, CT 06825, USA; boddend@mail.sacredheart.edu (D.B.); latesa@mail.sacredheart.edu (A.L.); anagnostn8@mail.sacredheart.edu (N.A.); moranm@sacredheart.edu (M.F.M.); 2 Department of Human Movement Sciences, Carroll University, Waukesha, WI 53186, USA; tsuchome@carrollu.edu

**Keywords:** bench press, ballistic, push-up, post-activation potentiation

## Abstract

The purpose of this study was to examine the effects of a ballistic or non-ballistic concentric-only bench press (COBP) on subsequent plyometric push-up performance. Fourteen resistance trained men completed two separate one-repetition-maximum (1RM) testing sessions followed by three randomized experimental explosive push-up sessions. These sessions combined a heavy concentric bench press with plyometric push-ups. Using a series of 3 × 10 (condition × time) repeated measures ANOVA, comparisons were made between the effects of ballistic and non-ballistic bench presses on performance of plyometric push-ups to investigate push-up performance variables. Compared with the control condition, both ballistic and non-ballistic bench presses produced lower net impulse and take-off velocity data. No differences were found between ballistic and non-ballistic conditions comparing net impulse and take-off velocity. We conclude that the magnitude of loading used in the current investigation may have caused acute fatigue which led to lower push-up performance characteristics. This information can be used to alter loading protocols when designing complexes for the upper body, combining the bench press and plyometric push-ups.

## 1. Introduction

A key performance outcome of a strength and conditioning program is the development of muscular power [1]. Power is derived from the intersection of force and velocity, both of which are modifiable in a resistance training plan. Many methods exist for the development of muscular power, which involve traditional resistance training, weightlifting movements, and plyometrics [2,3]. Another commonly used method of developing power is termed complex training. The goal of complex training is to pair biomechanically similar exercises in a sequence to take advantage of acute post-activation potentiation and increase the performance of subsequent movements [4,5]. For example, a number of studies have used squatting variations to enhance subsequent countermovement jump performance [6,7], while a number of upper-body protocols have been used to enhance ballistic throwing performance [8,9,10]. Regarding lower-body potentiation, several studies have demonstrated enhanced countermovement jump performance at various rest intervals [11,12,13], while other studies did not see improvements in performance [14,15]. Mixed results have also been displayed with upper-body potentiation protocols, with some studies showing an acute improvement in performance [8,9,10,16] and others showing no improvement or a decrement in performance [17,18].

Potential reasons for mixed results regarding potentiation may be due to the characteristics of the participants or the design of the potentiation complex itself [19]. Specific to the latter, the intent of the conditioning activity namely, the exercise stimulus completed before the subsequent activity may impact the timing and magnitude of potentiation. Compared to traditional resistance training, which has periods of acceleration and deceleration inherent to the movement, ballistic exercises provide a unique stimulus where athletes can attempt to accelerate through the whole range of motion [20,21,22]. This ability to alter acceleration may provide an avenue for improved performance by altering muscle activation, rate of force development, and neural drive [6,21]. A variety of ballistic exercises have been explored in both the upper and lower body for their ability to enhance sport performance through improvements in force, velocity, and power [6,8,10,13]. Previous investigations have examined both the force–time differences and performance outcomes of ballistic exercises on squatting and jumping performance [6]. These studies demonstrated that squatting with ballistic intent provided better force–time characteristics and enhanced jumping ability in trained male athletes. From a potentiation standpoint, additional research has demonstrated more rapid and larger magnitudes of potentiation following lower-body exercises performed with ballistic intent compared to the same exercises performed with non-ballistic intent [6,7,23].

While previous research has examined the differences in potentiation following lower-body exercises performed with ballistic and non-ballistic intent, limited research has examined these differences using upper-body exercises [6]. Given the interest in the use of complex training for both lower- and upper-body exercises in resistance training programs, it would appear that further research in this area is necessary. Therefore, the purpose of the current investigation was to examine the effects of ballistic or non-ballistic concentric-only bench press (COBP) exercises on plyometric push-up performance. We hypothesized that the ballistic condition would induce greater improvements in push-up performance compared to the non-ballistic condition. 

## 2. Methods

### 2.1. Experimental Approach to the Problem 

To explore the effects of ballistic or non-ballistic COBP on plyometric push-up performance, a repeated-measures design was implemented. Participants reported to the laboratory a total of five times for a measurement of two, one-repetition-maximum sessions (1RM) and three plyometric push-up conditions (control, ballistic, and non-ballistic). The first two sessions were separated by one week to prevent fatigue and the remaining three sessions were randomized in an attempt to prevent any effect of order on performance and were separated by 72–96 h. Participants were asked to refrain from any upper body training at least 72 h before any testing session to prevent any fatigue effects. A series of 3 (condition) × 10 (time) repeated-measures analyses of variance (ANOVA) were used to compare net impulse and take-off velocity between the different testing conditions.

### 2.2. Participants 

Fourteen men (mean ± SD; age = 21.0 ± 1.5 years, height = 174.5 ± 3.1cm, body mass = 77.7 ± 6.7 kg, bench press 1RM = 103.5 ± 18.3 kg, and bench press to body mass ratio = 1.3 ± 0.2) volunteered for this study. Participants were required to have resistance training experience for a minimum of one year, used a program that contained the bench press exercise, and be over the age of 18. Participants were excluded if they reported any orthopedic injuries in the past six months or did not complete the bench press on a frequent, regular basis. All participants read and signed a written informed consent form and all procedures were approved by the university’s Institutional Review Board. The Institutional Review Board approval number for this study is 171027A.

### 2.3. Procedures

#### 2.3.1. 1RM Bench Press Testing Session

The purpose of the bench press 1RM session was to determine the participants’ maximal bench press strength and record the safety bar height for the 1RM concentric-only bench press session. Before conducting the 1RM test, each participant completed a general dynamic warm-up. The warm-up consisted of 25 jumping jacks, 10 forward arm circles, 10 backward arm circles, 10 shoulder taps and 10 push-ups. Following the warm-up, two minutes of passive rest was given before initiating the 1RM test. The 1RM bench press test was implemented by adapting procedures from Suchomel et al. [6]. Participants performed submaximal warm-ups with repetitions at 30% (5 reps), 50% (3 reps), 70% (2 reps), and 90% (1 rep) of their self-reported 1RM. One minute of rest was given after repetitions at 30% and 50%, and 2 minutes after 70%. After the participants completed their last warm-up at 90%, 4 minutes of rest was given between all maximal attempts. Weights were determined and prescribed by the primary investigator based on self-reported 1RM data from the subject. A minimum 2.5 kg increase occurred between repetitions and participants were instructed to find their maximum in less than 5 total attempts. All repetitions were performed by lowering the bar to the sternum and completing the repetition to full lockout, confirmed by the research staff. Three spotters were present during all attempts to ensure safety and technical proficiency. 

Following the 1RM bench press session, the researchers determined the height of the safety pins for the next testing session for the 1RM concentric-only bench press. The laboratory assistants lowered the safety pins so that when the barbell was at the lowest position on the safety pin, it simultaneously rested on the participant’s sternum. This height for the safety pins was recorded and used for the remainder of the study.

#### 2.3.2. 1RM Concentric-Only Bench Press Testing Session and Familiarization 

One week following the 1RM bench press session, subjects reported to the laboratory at approximately the same time of day to determine their COBP 1RM from safety pins. The purpose of this session was to determine the weights that the participant would use under the experimental conditions and familiarize them with the concentric-only bench press from pins. The same general dynamic warm-up and 1RM protocol listed previously were used for this session. All repetitions of the COBP were performed with the barbell resting on the safety pins and the participant’s sternum. The participants then performed a COBP with full extension of the arms to complete each repetition. Each participant used progressively heavier loads, increased by a minimum of 2.5 kg for each attempt and a maximum was reached in less than 5 maximal attempts. 

**Figure 1:**
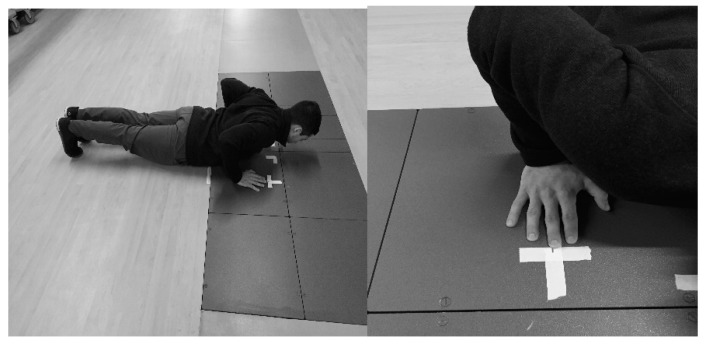
Start position hand placement for explosive push-ups.

After completing the 1RM and self-selected rest, participants practiced with each of the experimental conditions (ballistic and non-ballistic). During the ballistic condition, participants were instructed to “attempt to throw the bar but don’t let go” and were allowed 5 practice repetitions with a 20 kg barbell. During the non-ballistic conditions, participants were instructed to “press the bar” and were allowed 5 practice repetitions with a 20 kg barbell. Following the practice repetitions, the participant’s self-selected push-up hand placement on the dual force plates (2 separate 464 mm × 508 mm OPT464508 force plates; AMTI, Watertown, MA, USA) was determined. The distance from the top and side of the dual force plates to the distal end of the third digit was recorded and used for the remainder of the study. Hand placement and start position are shown in Figure 1. 

#### 2.3.3. Control Testing Session

Before starting the control testing session, participants completed the same general dynamic warm-up that was completed during the 1RM testing sessions. After completing the warm-up, participants were given one minute of passive rest before starting the plyometric push-up repetitions. Participants completed 1 explosive push-up with 1 minute of rest in between each repetition for 10 total trials. The participants were given the verbal cues “get set” where they placed their hands on the force plate in the bottom position and then received a countdown “3, 2, 1, up!”. Then, participants performed a plyometric push-up with their hands leaving the platform and then landing back onto the force plate under control. Participants completed a concentric-only plyometric push-up in this same manner for all trials. Consistent hand placement was ensured by using markers on the force plate based on the familiarization session. 

#### 2.3.4. Ballistic and Non-Ballistic Testing Sessions

The two experimental sessions and control sessions were randomized and completed in the same manner for all testing conditions. Following the same warm-up described above, participants completed a COBP potentiation protocol with ballistic or non-ballistic intent following 1 min of passive rest. The COBP potentiation protocol consisted of 5 repetitions at 30%, 3 repetitions at 50%, 3 repetitions at 70%, and 2 repetitions at 90% of the previously tested 1RM COBP. Participants rested for one minute between 30% and 50%, 2 minutes between 50% and 70%, and 3 min between 70% and 90%. Participants were given verbal encouragement on all repetitions based on the script detailed above. After the final COBP repetition, one minute of rest was provided prior to the participant performing 10 plyometric push-ups on the dual force plates in the same manner as the control testing session. 

### 2.4. Data and Statistical Analysis 

All push-up repetitions were performed on dual force plates sampled at 1000 Hz. The data were collected using NetForce software (vers. 9.4.0.813654; AMTI, Watertown, MA, USA). All data were analyzed using a custom written MATLAB program (The Mathworks Inc., Natick, MA, USA). 

Normality of the data was assessed using the Shapiro–Wilk test. Test–retest reliability of net impulse and take-off velocity was assessed using the control condition data with two-way mixed intraclass correlation coefficients. To examine the potential differences between the control, ballistic, and non-ballistic conditions, two sets of 3 (condition) × 10 (time) repeated measures ANOVA were used. If statistical significance was present for the main effect or interaction data, Bonferroni post hoc analyses with 95% confidence intervals (CI) were used. If the assumption of sphericity was violated, Greenhouse–Geisser adjusted values were used. Practical significance at each time interval between conditions was assessed using Cohen’s d effect sizes. Effect size magnitudes of 0–0.19, 0.20–0.59, 0.60–1.19, 1.20–1.99, 2.0–3.99, and ≥4.0 were interpreted as trivial, small, moderate, large, very large, and nearly perfect, respectively [24]. All statistical analyses were performed with SPSS (version 25, IBM Armonk, NY, USA), using *p* ≤ 0.05 as a standard of statistical significance.

## 3. Results

Net impulse and take-off velocity data were normally distributed. Test–retest reliability of the net impulse and take-off velocity data ranged from 0.95 to 0.96.

### 3.1. Condition Main Effects

A statistically significant main effect of the conditions existed for both net impulse (*p* < 0.001, power = 0.99) and take-off velocity (*p* < 0.001, power = 0.99). Post hoc analysis indicated that the control condition produced greater time-averaged net impulse characteristics compared to the ballistic (*p* = 0.004, CI = 2.21–11.85) and non-ballistic conditions (*p* = 0.013, CI = 1.51–13.35). In contrast, no statistical differences existed between the ballistic and non-ballistic conditions (*p* = 1.000, CI = −2.90 to 3.71). Similar to net impulse, post hoc analysis indicated that the control condition produced greater time-averaged take-off velocities compared to both the ballistic (*p* = 0.005, CI = 0.04–0.23) and non-ballistic conditions (*p* = 0.011, CI = 0.03–0.26). No statistical differences existed between the ballistic and non-ballistic conditions for time-averaged take-off velocity (*p* = 1.000, CI = −0.06 to 0.08).

### 3.2. Time Main Effects

A statistically significant main effect of time existed for both net impulse (*p* < 0.001, power = 0.98) and take-off velocity (*p* < 0.001, power = 0.99). Post hoc analysis indicated that the condition-averaged net impulse produced at minute 2, post-stimulus, was statistically greater than the magnitude produced at minute 9, post-stimulus (*p* = 0.019, CI = 0.61–10.53). However, no other statistically significant differences existed between time intervals (*p* > 0.05). In reference to condition-averaged take-off velocity, the magnitude produced at minute 1, post-stimulus, was statistically greater than the magnitude produced at minute 9, post-stimulus (*p* = 0.044, CI = 0.00–0.43). Similarly, the take-off velocity produced at minute 2, post-stimulus, was statistically greater than the magnitude produced at minute 9, post-stimulus, (*p* = 0.016, CI = 0.01–0.19). No other statistically significant differences existed between time intervals (*p* > 0.05).

### 3.3. Condition x Time Interaction Effects

**Figure 2:**
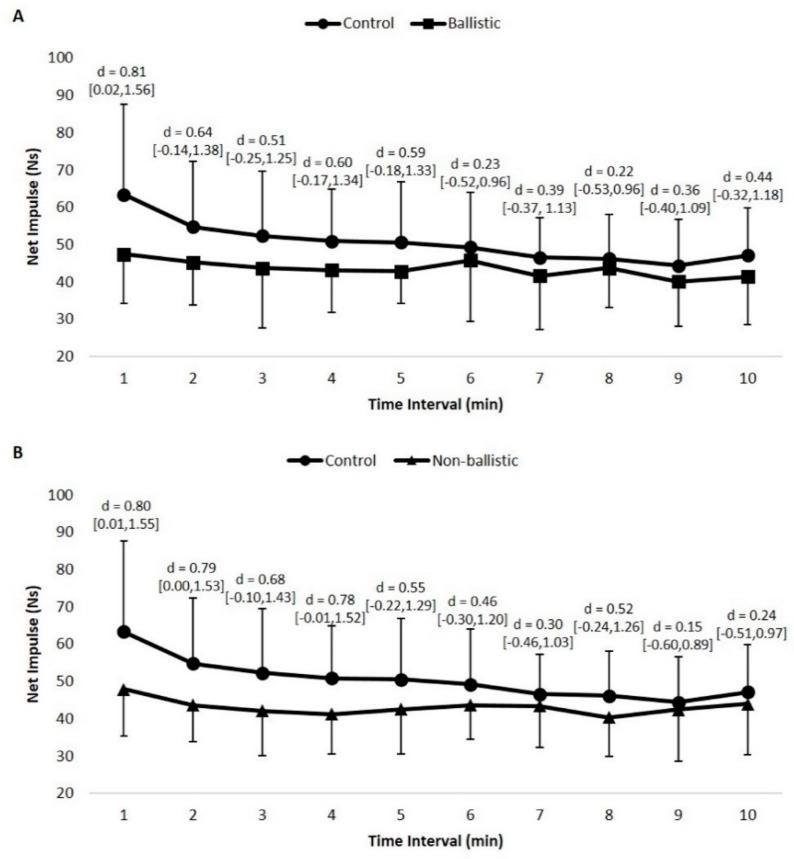
Effect size comparisons for net impulse at each time interval between the control and ballistic conditions (**A**) and control and non-ballistic conditions (**B**); 95% confidence intervals are reported within the brackets.

**Figure 3:**
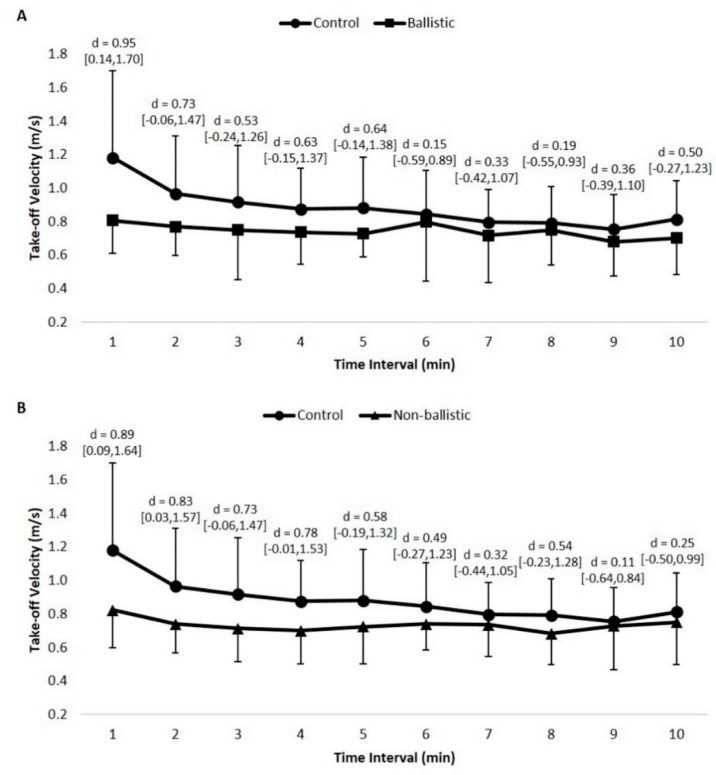
Effect size comparisons for take-off velocity at each time interval between the control and ballistic conditions (**A**) and control and non-ballistic conditions (**B**); 95% confidence intervals are reported within the brackets.

Statistically significant condition x time interaction effects existed for both net impulse (*p* = 0.035, power = 0.95) and take-off velocity (*p* = 0.005, power = 0.99). The effect size differences between the control condition and the testing condition at each time interval are presented in Figure 2 and Figure 3. Post hoc analysis of net impulse revealed statistically significant differences within the control condition (*p* = 0.003), but not for the ballistic (*p* = 0.269) or non-ballistic conditions (*p* = 0.080). Specifically, net impulse at 2 (*p* = 0.046, *d* = 0.68) and 4 minutes (*p* = 0.024, *d* = 0.49), post-stimulus, was statistically greater than the net impulse at 9 minutes, post-stimulus, while no other statistically significant differences existed (*p* > 0.05) during the control condition. It should be noted that the effect sizes ranged from trivial to moderate compared to the lowest net impulse at 9 min, post-stimulus (*d* = 0.14–0.98), dependent on the rest interval. Similar to net impulse, post hoc analysis revealed statistically significant differences within the control condition for take-off velocity (*p* = 0.005), but not for the ballistic (*p* = 0.342) or non-ballistic conditions (*p* = 0.178). A specific difference included a greater take-off velocity at 4 minutes, post-stimulus, compared to 9 min, post-stimulus (*p* = 0.036, *d* = 0.53); however, no other differences between rest intervals existed (*p* > 0.05). It should be noted that effect sizes compared to the slowest take-off velocity ranged from trivial to moderate (*d* = 0.18–1.08), dependent on the rest interval.

## 4. Discussion

The purpose of this investigation was to compare the acute effect of ballistic and non-ballistic COBP on subsequent plyometric push-up performance. Specifically, the authors endeavored to compare ballistic with non-ballistic COBP on net-impulse and take-off velocity in explosive push-ups. The main outcomes from this study indicated that, compared to a control session, both experimental conditions produced smaller magnitudes of net impulse and take-off velocity, while no differences existed between the ballistic and non-ballistic conditions. 

Prior research investigating upper-body potentiation complexes has demonstrated improvements in upper-body explosive performance [8,9,10,16]. The current investigation could not replicate such findings in the upper body combining a bench press and plyometric push-ups. Specifically, the current study showed decrements in net impulse and take-off velocity following ballistic and non-ballistic COBP repetitions. These outcomes may have occurred due to the acute fatigue produced by the heavy COBP. Previous research has indicated that both fatigue and potentiation are present following a pre-conditioning stimulus; however, whether or not potentiation is realized is based on which magnitude is larger [25]. Baker [8], noted that the relative strength of the participants may affect the timing and magnitude of potentiation. While the average bench press relative strength of the current study population was 1.3 x their body mass, the strength levels ranged from 1.04 to 1.67. The potentiation protocols in the current study concluded with two repetitions at 90% of the COBP 1RM. While stronger individuals may have been able to resist fatigue at this load, weaker individuals may not have. Thus, it is possible that any potentiation effects that were present were masked by some of the weaker participants in the current study. However, given the paucity of research, it is important that future research investigate the relationship between muscular strength and upper-body potentiation. 

In a series of lower-body studies, Suchomel et al. [6,7,26], demonstrated that ballistic concentric-only half-squats could be used to potentiate squat jump performance. Similarly, West et al. [9] demonstrated enhanced bench press throw power output. The results of the current study are in contrast to the previous studies as the ballistic condition showed no difference in performance compared to the non-ballistic condition, and actually displayed a decrement in performance compared to the control condition. There are several potential reasons for these findings. First, as mentioned above, the load used in the current study to potentiate the explosive push-ups was 90% of the 1RM COBP. This load was much larger than the ballistic load used by West et al. [27], which was 30% of the 1RM and may have contributed to greater levels of fatigue and masked any potentiation effects that may have been present. Second, and in contrast to West et al. [9], the ballistic condition in the current study did not have the individuals throw the barbell. Previous research has indicated that bench press throws, where the individual loses contact with the barbell, may result in greater muscle activation, rate of force development and neural drive compared to bench press repetitions performed quickly [20]. Because the participants in the current study were instructed not to lose contact with the barbell at the end of their ballistic condition repetitions, it is possible that a deceleration period occurred, which may have prevented the nervous system from being stimulated to the point of producing a potentiated state. However, because the current study did not measure muscle activation, it is suggested that future research investigate this relationship. Finally, the current study used COBP repetitions to enhance an explosive push-up performed from a static start. This was done from a specificity standpoint and followed a similar methodology used in a series of lower-body potentiation studies [6,7,26]. From a practical standpoint, explosive push-ups are typically performed using a stretch-shortening cycle action. West et al. used a stretch-shortening cycle action when they performed their ballistic bench press throws [9]. Previous research has demonstrated that a stretch-shortening cycle action contributes to greater force, power, and neural drive compared to concentric-only upper-body movements [20]. Specifically, the authors noted that the upper-body musculature may be limited in its ability to produce force during fast contraction velocities of concentric-only movements. Whether it was based on relative strength level or their muscle characteristics (e.g., fiber type, muscle mass, etc.), it appears that the participants in the current study, on average, were unable to benefit from a large neural stimulus placed on their nervous system. This may have prevented them from displaying a potentiated performance during their plyometric push-ups.

This study had several limitations to consider. First, there was a relationship between strength levels and acute performance outcomes after heavy loading [7]. The participants in the study were resistance-trained and possessed on average a relative strength level of 1.3, but some of the weaker participants may have acquired more fatigue than the stronger participants in this investigation. Secondly, participants were of a heterogeneous training background which meant that some participants may have had a lack of plyometric training prior to the study, possibly altering the response to the push-up task. Future studies should attempt to use trained, strong athletes with a history of upper-body plyometric exercises to further elucidate the effect of upper-body complexes.

## 5. Conclusions

The results of the present investigation indicated that heavy COBP preceding plyometric push-ups did not elicit a performance improvement. Furthermore, performing the bench press in a ballistic or non-ballistic manner did not alter any of the measured performance variables. Although performance outcomes were not obtained from this loading prescription, future studies should examine the effects of a lighter magnitude of loading prior to plyometric push-ups. Finally, coaches should be aware that loading prescription used for lower-body muscle groups may differ from the muscles of the upper-body and may necessitate alterations in a training protocol.
